# Fungi Associated with Olive Tree (cv. ‘Nocellara del Belice’) Decay in Trapani Province (Sicily, Italy)

**DOI:** 10.3390/pathogens13110932

**Published:** 2024-10-25

**Authors:** Marika Lamendola, Giulia Mirabile, Josè Muratore, Livio Torta

**Affiliations:** 1 Dipartimento Scienze Agrarie, Alimentari e Forestali, Università degli Studi di Palermo, Viale delle Scienze Ed. 5, 90128 Palermo, Italy; marika.lamendola@unipa.it (M.L.); livio.torta@unipa.it (L.T.); 2 Ispettorato Provinciale dell’Agricoltura di Palermo, Regione Sicilia, Via Camillo Camilliani 54, 90145 Palermo, Italy; 3 Ente Sviluppo Agricolo, Servizi allo Sviluppo, Regione Sicilia, Via Libertà 203, 90143 Palermo, Italy; jo.muratore@gmail.com

**Keywords:** *Olea europaea*, decay syndrome, *Coriolopsis gallica*, *Fomitiporia mediterranea*, *Pleurostoma richardsiae*, *Kirschsteiniothelia*, Sicily

## Abstract

Recently, in several locations in the province of Trapani (Sicily, Italy), olive growers have reported cases of decaying olive trees of cv. ‘Nocellara del Belice’, showing symptoms of defoliation, branch drying, xylem browning, and reduced production. Internal symptoms include white and brown wood rot, starting from the base of the trunk. These alterations have been observed in trees irrigated using a pipe system at the trunk with spray sprinklers. To identify the causal agents of decay, some trees were eradicated and dissected, and woody samples were processed to isolate and identify the associated fungal micro-organisms. The most common colonies were identified using morphological (macro- and microscopical observation) and molecular (PCR amplification of the rDNA-ITS region) analyses. Nine fungal taxa were identified, of which four were associated with this decay syndrome (*Coriolopsis gallica*, *Fomitiporia mediterranea*, *Kirschsteiniothelia* sp., and *Pleurostoma richardsiae*), three were considered ubiquitous and opportunistic fungi (*Alternaria* spp., *Aspergillus amstelodami*, and *Trichoderma* sp.), and the other two were *mycelia sterilia*. Artificial inoculation satisfied Koch’s postulates, confirming the pathogenicity of the aforementioned fungi, even though the infections in the fields seem to be related to the irrigation system. This hypothesis would seem to be confirmed by the progression of decay over time in the trees subjected to the irrigation system described but not reported in olive groves differently managed. It is therefore considered appropriate to conduct further and more in-depth investigations aimed at studying the correlation between the irrigation system, presence of fungal agents, and manifestation of the syndrome. A further ongoing investigation is aimed at the use of biostimulants (Agrusaver, Savory Sun, VA LLC) on symptomatic trees, with the aim of both improving the vegetative performance of the host and limiting the symptoms detected in the field.

## 1. Introduction

The olive tree (*Olea europaea* L.) is one of the most representative botanical species in the Mediterranean basin. For millennia, it has been present as a cultural element in artistic, literary, and religious expression due to its symbolic value. Moreover, it is a traditional crop in the Mediterranean region, cultivated since ancient times and dispersed across a wide area. The biological lifespan of olive trees is hundreds of years, but it does not usually correspond to the duration of an olive grove’s economic viability; in fact, modern intensive or highly intensive cultivation methods have drastically reduced their crop cycle [1]. 

Many diseases can affect olive trees, their fruit, leaves, branches, trunks, collars, and roots (or the entire host), and common biotic etiological agents include viruses, phytoplasmas, bacteria, fungi, and nematodes [2,3]. The determination of both abiotic and biotic decay agents leads to the development of specific strategies, aimed to reduce damages in trees [4,5,6]. Recently, in all areas of olive tree cultivation, there have been both well-known and newly reported occurrences of olive tree decay in association with phytopathogenic fungi (*Botryosphaeria* spp, *Phaeoacremonium* spp., *Phaeomoniella* sp., *Phoma incompta*; *Stereum hirsutum*, and *Verticillium* spp.). Specifically, in Brazil, China, Croatia, Greece, Italy, Spain, Thailand, and California and other locations in the United States, syndromes characterized by both external and internal symptoms, such as xylem browning and white and brown rot, have been described and studied, including the identification of the respective etiological fungal agents (*Botryosphaeria* spp.—olive branch dieback, canker, and decline; *Phaeoacremonium* spp. and *Phaeomoniella* sp.—rapid dieback of shoots, twigs, and branches; dark yellowing and browning of the leaf tips; and death of the olive plant; *Stereum hirsutum*—wood rotting of olive trees) [7,8,9,10,11,12,13,14,15,16]. Some of these fungal agents, such as *Botryosphaeria* spp., affect many species; in fact, they are important pathogens of pome and stone fruit trees, causing fruit rots (e.g., black and white rot of apple), frogeye leaf spot, stem and branch cankers, gummosis, dieback, and in some cases, tree death [17]. In many cases, predisposing causes, such as defective irrigation systems, prolonged drought, and other abiotic factors, weaken the host, increasing its susceptibility even to pathogens that are not particularly aggressive (*Alternaria alternata*, *Alternaria consortialis*, *Arthrinium phaeospermum*, *Berkeleyomyces basicola*, *Botrytis cinerea*, *Colletotrichum acutatum*, *Dothiorella iberica*, *Epicoccum nigrum*, *Fusarium oxysporum*, *Macrophomina phaseolina*, *Neocosmospora solani*, and *Neofabraea vagabunda*) [18]. Similar processes have been described in many forest trees [19]. 

Olive tree decay must be considered a global phytopathological emergency to monitor, and studies have aimed to define the agents that are directly or indirectly related to the evolution of the disease to mitigate the causes of disturbance and thus restore the best vegetative conditions.

Within the varietal range of table olive tree cultivars in western Sicily, cv. ‘Nocellara del Belice’ is one of the best known and most widespread. This variety excels in oil production, meeting high quality and quantity standards. In cultivation, olive grove management varies according to the destination of the product: the oil mill or canning industries. One of the main management differences concerns irrigation techniques. To obtain large-sized, well-preserved, and highly prized table fruit, abundant watering is required in the warmer periods, especially in July and August. To guarantee a localized water supply that reduces waste and, at the same time, provides the necessary supply, new irrigation systems have been utilized since the 1990s.

**Figure 1:**
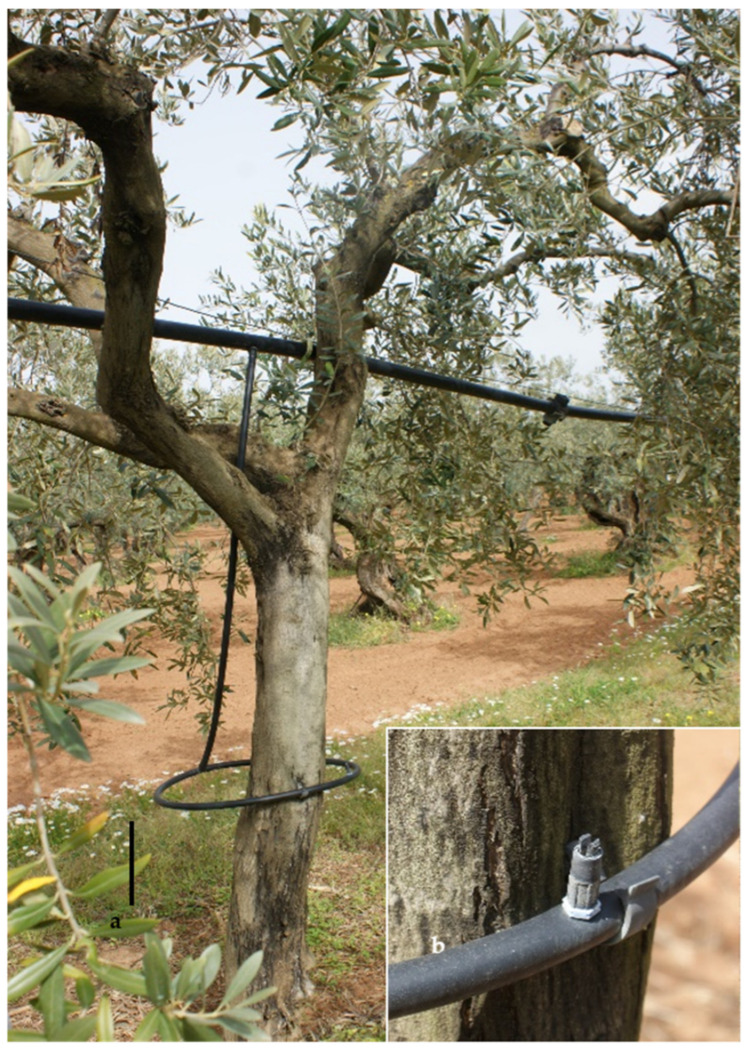
Irrigation system applied in the monitored olive groves: circular collar around the trunk ((**a**); bar = 30 cm) and close-up of the sprinkler ((**b**); bar = 3 cm).

At the beginning of 2021, several olive growers in the vicinity of Campobello di Mazara, Castelvetrano, Partanna (Trapani Province, Sicily, Italy), reported cases of decay associated with xylem alterations in 30–40-year-old cv. Nocellara del Belice olive trees destined for table olive production. In these groves, the trees were equipped with a sprinkler irrigation system under the foliage, with a circular collar around the trunk (Figure 1a) and a 180° micro-sprinkler (Figure 1b). Until now, this problem has not existed in Sicily, as the plants had apparently been healthy until 2021, when olive growers observed the appearance of the first symptoms.

On this basis and in collaboration with the Agricultural Development Agency of the Region of Sicily, an investigation aimed at identifying the causes of the reported decay was initiated using isolation assays and through further identification of the associated fungal micro-organisms. Their pathogenicity was subsequently evaluated with artificial inoculation tests. The results of this study create the basis for conducting further investigations aimed at using strategies that can limit the progression of the disease in the field and prevent the presence of the disease in new olive groves.

## 2. Materials and Methods

### 2.1. Monitoring and Sampling

Based on reports from local olive growers, the phytosanitary status of the trees was monitored by sampling symptomatic and asymptomatic tree tissues for laboratory analyses. In the time frame from March 2022 to April 2023, 10 trees (in total), 8 symptomatic and 2 asymptomatic, were selected from 6 olive farms. For each tree, 3 trunk disks with a thickness of approximately 10 cm were collected at the collar (basal portion, B), at a height of 80 cm (median portion, M) and 150 cm (apical portion, A). The samples were labeled, each with their respective code (tree number and portion) and transported to the Plant Pathology Laboratories of the SAAF Department of the University of Palermo (Palermo, Sicily, Italy) for analyses.

### 2.2. Isolation of Fungi

To isolate the fungi associated with trunk symptoms, samples were cut crosswise and lengthwise and surface-disinfected by flambéing. For each sample, 15 xylem chips from the margin of the chromatic alteration were aseptically collected using a sterile hammer and chisel, and 3- × 3-mm fragments were placed onto potato dextrose agar (PDA, Oxoid, UK; 3 Petri dishes per portion and 5 fragments per Petri dish). All plates were incubated at 24 °C in the dark for 3 weeks and observed every 3 days. The fungal colonies were counted, observed by the naked eye and under a stereomicroscope (Zeiss, Oberkochen, Germany; 47 50 52-9901), and grouped into morphotypes. The isolation frequency of the fungal colonies (IF) was calculated using the formula IF = (Nif/Nft) × 100, where Nif is the number of colonies and Nft is the total number of isolations attempted × 100. Based on morphotype and preliminary identifications, the most frequently isolated fungal colonies were transferred onto new PDA by transferring PDA agar plugs of actively growing mycelium (7 mm in diameter) first onto new PDA plates and then into tubes and maintained on PDA at 4 °C [20].

### 2.3. Morphological and Cultural Characterization of Isolates

The microscopic features of the isolated colonies were observed under a light microscope (Axioscope Zeiss, Oberkochen, Germany) coupled with an AxioCam MRc5 digital camera (Zeiss, Oberkochen, Germany). For each isolate, the sizes of the fungal structures (such as spores, conidia, fruiting bodies, and conidiophores) and other taxonomic characteristics were calculated as the arithmetic mean of 30 measurements, and images were viewed and saved using the AxioVision 4.6 software (Zeiss, Oberkochen, Germany) [20,21]. Slides were mounted with a drop of lactophenol, clear, or methyl blue (0.01%) containing small masses of mycelial colonies and covered with a cover glass. At the end of the incubation period (9 days at 24 °C in the dark), colony characteristics (color, mycelium, colony type, and shape) were observed [22,23,24,25]. Available conidia and hyphal features (color, shape, and presence or absence of septa and clamps) were also recorded. To evaluate and describe the microscopic features of the colonies, actively growing mycelia from culture margins were prepared, and microscopic observations were carried out.

### 2.4. Sequencing of Internal Transcribed Spacer Regions

To molecularly characterize fungal isolates, the internal transcribed spacer (ITS) region, β-tubulin (Bt), elongation factor (EF), and calmodulin (CMD) genes of ribosomal RNA were amplified. Fungal DNA was extracted from little mycelial mass, sterilely taken by a needle from pure fungal colonies, using the Extract-N-Amp™ DNA Extraction Kit (Sigma-Aldrich, St. Louis, MO, USA), following the manufacturer’s instructions. 

For amplification, PCR ReadyMix containing buffer, salts, dNTPs, Taq polymerase and JumpStart™ Taq antibody was used together with the primers. The primers used were ITS1 (5′-CTTGGTCATTTAGAGGAAGTAA-3′) and ITS4 (5′-TCCTCCGCTTATTGATATGC-3′) [9,26], EF1-728F (5′-CATCGAGAAGTTCGAGAAGG-3′) and EF1-986R (5′-TACTTGAAGGAACCCTTACC-3′), Bt2a (5′-GGTAACCAAATCGGTGCTGCTTTC-3′) and Bt2b (5′-ACCCTCAGTGTAGTGACCCTTGGC-3′), CMD5 (5′-CCGAGTACAAGGARGCCTTC-3′), and CMD6 (5′-CCGATRGAGGTCATRACGTGG-3′). All PCR assays were carried out in a MultiGene OptiMax thermocycler (Labnet International, Edison, NJ, USA). PCR was performed using the following settings: ITS and Bt primers: denaturation at 95 °C for 2 min, followed by 35 cycles of 60 s at 95 °C, 60 s at 55 °C, and 60 s at 72 °C, and final extension at 72 °C for 5 min; EF and CMD primers: denaturation at 95 °C for 3 min, followed by 35 cycles of 30 s at 95 °C, 30 s at 50 °C, and 30 s at 72 °C. Amplified products were separated by agarose gel electrophoresis at a concentration of 1.5% in 1X TBE at 5.6 V cm^−1^. The PCR products were sent to BMR Genomics Srl (Padova, Italy) for sequencing.

### 2.5. Phylogenetic Analyses

The obtained sequences, manually edited when needed, were compared with the sequences present in GenBank using the Basic Local Alignment Search Tool (BLASTn) of the National Center for Biotechnology Information (NCBI; USA) and deposited in GenBank. A phylogenetic analysis was conducted only on the sequences obtained using the ITS primers. Sequences with 99–100% similarity and other representative sequences obtained in previous studies [27,28,29] were retrieved from GenBank and aligned with those obtained in this study. Alignments were performed using Clustal W 1.81, and a neighbor-joining starting tree was generated using MEGA11. *Polyporus tricholoma* (AF516555.1), *Phellinus uncisetus* (GU461960.1), and *Diaporthe ambigua* (NR119434.1) were selected as outgroup taxa, and 1000 bootstrap replicates were performed. 

### 2.6. Pathogenicity Tests

To confirm their pathogenicity, the identified fungal taxa, reported in the literature as being associated with symptoms similar to those detected in the field, were used to inoculate healthy Nocellara del Belice trees. 

**Figure 2:**
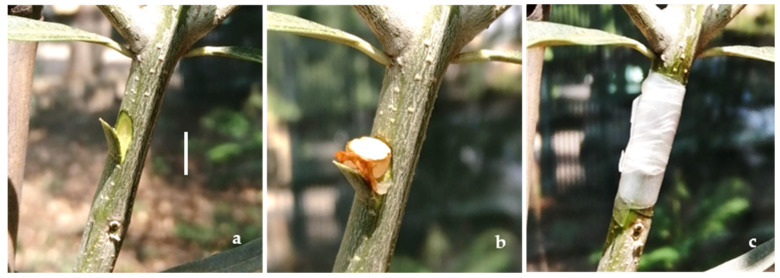
Artificial inoculation: wounds in the basal, medial, and apical portions of the stem (**a**); insertion of a micellar disk taken from the fungal culture (**b**); wound sealing with parafilm (**c**). (**a**–**c**): Bar = 5 mm.

In April 2023, sixteen 2-year-old trees were inoculated with 4 fungal species (4 trees per species). Artificial inoculation was carried out by making 5.5 mm × 10 mm wounds in the basal, medial, and apical areas of the stem using a sterile scalpel, inserting one 5.5 mm diameter mycelial plug taken from the margin of a 15-day-old PDA culture, sealing the wound with parafilm, and fastening the area with paper tape to protect the inoculum (Figure 2a–c). Four additional olive trees were inoculated with sterilized PDA (controls). The trees were placed under natural conditions in an experimental field adjacent to the SAAF Department. Two trees per species were visually checked at 30 and 180 days after inoculation. Re-isolation was performed on symptomatic tissues, and the isolates were identified, as described previously, to satisfy Koch’s postulates [10,12,30,31].

## 3. Results

### 3.1. Identification and Phylogenetic Analyses of Fungal Isolates

**Figure 3:**
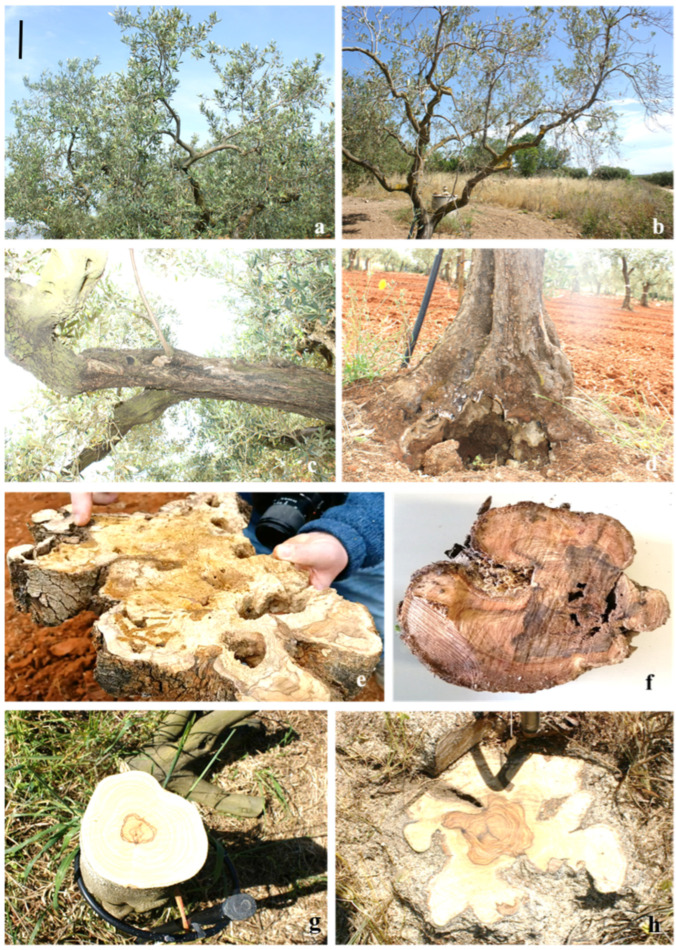
External symptoms: foliar chlorosis (**a**), defoliation (**b**), branch drying (**c**), and reduced anchoring (**d**); internal symptoms of symptomatic trees: browning and white (**e**) and brown rot (**f**); internal symptoms of asymptomatic trees: browning (**g**) and brown rot (**h**). Bar: (**a**,**b**) = 50 cm; (**c**,**d**) = 20 cm; (**e**–**h**) = 10 cm.

The decaying trees showed external symptoms, such as small leaves, foliar chlorosis, defoliation, shoot and branch drying, wood rot, cankers, reduced anchoring, flowering, and fruiting (Figure 3a–d). The sectioned branches showed browning, and in the basal area, there were extensive portions of white and brown rot (Figure 3e,f). The same internal symptoms were also observed in asymptomatic trees (Figure 3g,h). 

The growth of fungal colonies from the woody fragments was detected up to 9 days after the assays were set up, and from the 10th day, no growth of new colonies was observed. The presence of fungal micro-organisms was observed in all tested trees, except for symptomatic tree no. 3. Of the 450 wood fragments, 117 produced fungal colonies (26%), of which 46% were distributed at the trunk base, 27% in the median part, and 27% in the apical portion. In terms of the distribution of fungal entities in the trees under study, the number of isolates obtained ranged from 1 to 29 (Table 1). Based on the morphological characteristics of the isolated colonies, nine morphologically similar groups were distinguished (Table 1).

The trees from which the colonies were isolated are indicated below: *Altenaria* spp. and *Aspergillus* sp. from trees number 1, 5, 6, 7, and 8; *C. gallica* from trees number 5 and 7; *F. mediterranea* from trees number 2, 4, and 7; *Mycelia sterilia* 1 from trees number 1, 2, 5, 6, 7, and 8; *Mycelia sterilia* 2 from trees number 9 and 10; *P. richardsiae* from trees number 2 and 6; *Trichoderma* sp. from trees number 1, 5, 6, 7, and 8.

**Figure 4:**
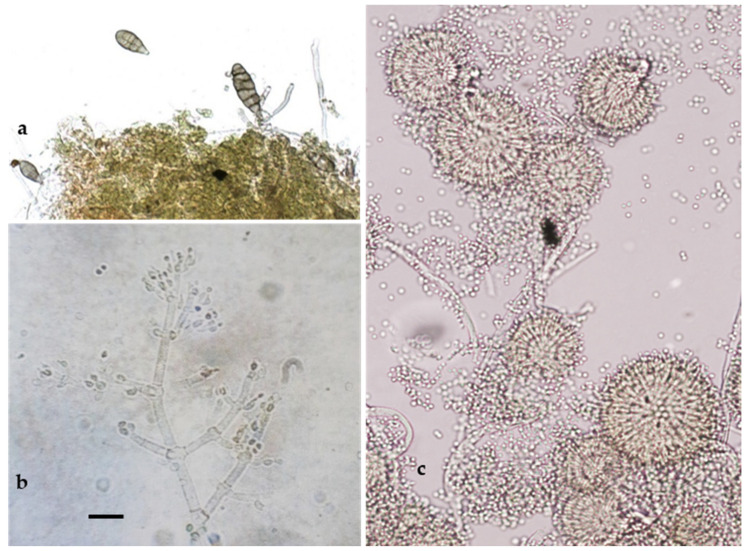
Anamorphic stages of *Alternaria* sp. (**a**); *Trichoderma* sp. (**b**); and *Aspergillus amstelodami* (**c**). Bar = 20 µm.

As reported in Figure 4, the microscopic observations allowed us to detect some taxonomic characteristics (shape and size of conidia and conidiophores) typical of the genera *Alternaria* Nees (14%; Figure 4a), *Aspergillus* Link (7%; Figure 4b), and *Trichoderma* Peers (9%; Figure 4c), which are known as contaminating, ubiquitous, and opportunistic fungi.

**Figure 5:**
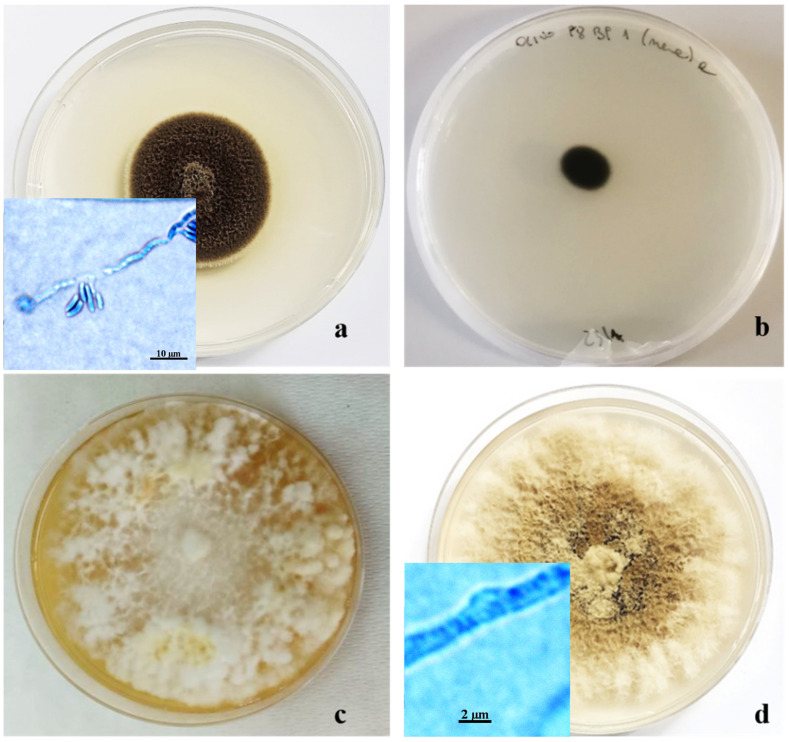
Ten-day-old colonies of *Pleurostoma richardsiae* (**a**); *Kirschsteiniothelia* sp. (**b**); *Fomitiporia mediterranea* (**c**); and *Corolopsis gallica* (**d**) grown in Petri dishes (Ø 10 cm) on PDA.

One morphotype, making up 10% of the isolated fungal colonies, was characterized by a cottony appearance, initially white in the center but becoming darker at the margin, ranging from gray to brownish black. The conidia, of two different types, were globular, dark, 1.5 µm in diameter, and with hyaline cylindrical allantois (6–6.5 × 2 µm). The phialides were lateral or terminal, with hyaline collars 1–2 µm long and 0.5–0.9 µm wide. This set of morphological characteristics led us to consider these colonies as belonging to the *Pleurostoma* genera (Figure 5a) [12,13,22,32,33].

Another morphological group, consisting of 10% of the isolated colonies, was characterized by very dark colonies with compact mycelium and grazing of the substrate, consisting of septate, branched, and smooth-walled hyphae. On PDA, these colonies did not produce conidia or other structures useful for taxonomic identification (Figure 5b).

Two other morphotypes, both represented by fungal colonies with sterile mycelium, showed the presence of hyphae with clamp unions, which are typical of basidiomycetes, under a light microscope. The largest group, consisting of 18% of isolates, showed floccose, dense mycelium, initially whitish, and then creamy yellow, up to amber, on PDA (Figure 5c). The other group, representing 8% of the isolates, included colonies characterized by felty, grayish mycelium, tending to brown when ripe (Figure 5d). Molecular analyses, based on the amplification products of the ITS region, confirmed morphological identification at the genus level. The sequence obtained showed 99–100% similarity with reference strains on GenBank, and each strain was deposited with its accession number. Some of the other primers used gave positive results until the electrophoretic run on agarose gel; then, sequencing was not clean. Thus, it was not possible to proceed with the comparison with GenBank, while other primers did not provide any results in the electrophoretic run on agarose gel (Table 2).

The *Aspergillus* colonies were identified as *Aspergillus amstelodami* (Mangin) Thom et Church, while others were found to belong to the genus *Pleurostoma* as *Pleurostoma richardsiae* (Nannfeltd) (syn. = *Pleurostomophora richardsiae*; accession number OP028963). Furthermore, isolates of the morphotype with blackish mycelium were identified as belonging to *Kirschsteinothelia* D. Hawksw., while the floccose yellowish colonies and the grayish-brown ones (Figure 5c,d) belonged to basidiomycetes *Fomitiporia mediterranea* M. Fisch (OP028964) and *Coriolopsis gallica* (Fr.) Ryvarden (OP028965), respectively. Analyses aimed at identifying the two morphological groups with sterile mycelium are currently underway. 

**Figure 6:**
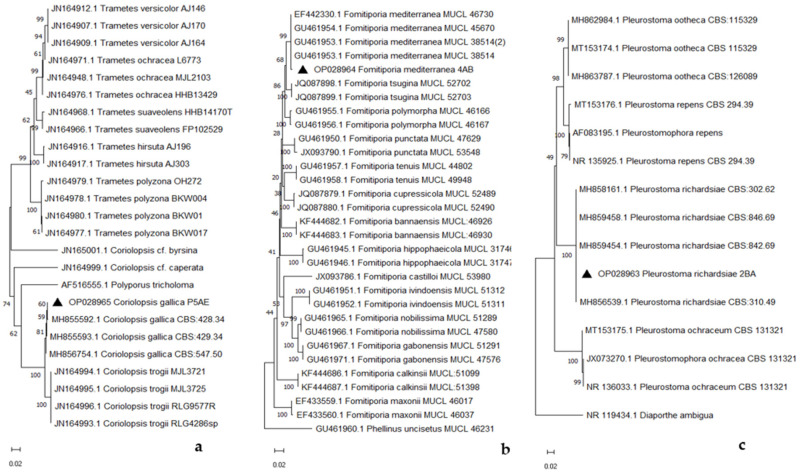
Phylogenetic tree of *Coriolopsis gallica* (**a**), *Fomitiporia mediterranea* (**b**), and *Pleurostoma richardsiae* (**c**).

Phylogenetic analysis, based on ITS sequence data, confirmed the taxonomic placement of the strains and showed that our isolates (▲ in Figure 6a–c) could be grouped with strains of *C. gallica*, *F. mediterranea*, and *P. richardsiae* identified in other studies [10,34,35].

### 3.2. Pathogenicity Assays

**Figure 7:**
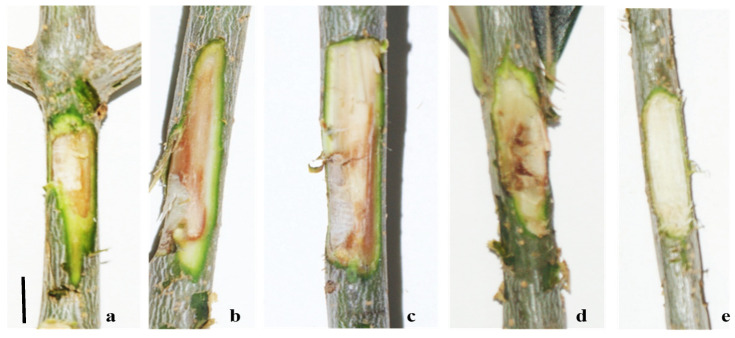
Results of artificial inoculation with *Fomitiporia mediterranea* (**a**); *Kirschsteiniothelia* sp. (**b**); *Pleurostoma richardsiae* (**c**); *Coriolopsis gallica* (**d**); and control (**e**). Bar: (**a**,**e**) = 1 cm; (**b**–**d**) = 5 mm.

Pathogenicity tests were carried out by inoculating with one isolate of *F. mediterranea*, *P. richardsiae*, and *C. gallica*, which have been reported in the literature as possible agents of xylem alterations. A colony of *Kirschsteinothelia* sp., which is lacking in the literature data, was also used to acquire knowledge of its potential pathogenicity. The other fungal strains identified were not used in the pathogenicity assays, as, in the literature, they are not associated with the symptoms described in the field and are considered ubiquitous and opportunistic fungi. The first observation conducted on two trees began 30 days after inoculation (1) [10], corresponding with visible browning at the injection point (Figure 7). At the time of sampling for laboratory analysis after artificial inoculation, xylem lesions extended approximately 2 cm above and 1 cm below the inoculation point. *Fomitiporia mediterranea, Kirschsteiniothelia* sp., and *P. richardsiae* were re-isolated from artificially inoculated trees, confirming their pathogenicity. Six months after artificial inoculation, *C. gallica* was also re-isolated, satisfying Koch’s postulates. 

## 4. Discussion and Conclusions

In recent years, widespread dispersal of xylem diseases has been observed throughout the world in cultivated, ornamental, and wild tree species. This increase appears to be due to various factors, such as unfavorable vegetative conditions following climate change, serious nutritional and irrigation imbalances, stress from pollution, and attack from new or more virulent biotic agents [1]. Our investigation permitted the identification of some fungal micro-organisms associated with decay in “young” cv. Nocellara del Belice olive trees, cultivated with a specific strategy for producing table olives in a well-defined territory of Trapani. The basidiomycetes *C. gallica* and *F. mediterranea* have been reported in the literature as agents of rot and decay of various tree species.

*Coriolopsis gallica* is a common species in Italy, less frequent in Northern Europe, more frequent in Southern Europe, and quite widespread in North America, although it is not particularly aggressive. The fruiting bodies are annual, sessile, dimidiate, often with the margin folded, imbricated, and rarely resupinated. They can reach a length of 10–15 cm, a width of 5–7 cm, and a thickness of 1–1.5 cm. The upper surface is tomentose, zoned, and covered with dense tufts of adherent brown hyphae, which are grouped together, with colors ranging from brown to reddish to ochre. The poroid surface is brown and dark in adult specimens, if it is touched or rubbed. The pores are irregular, angular, and extend 1–2 (3) per mm. The dissepiments are initially whole and thick, covered by a thin bloom, and then become thin and torn. The margins are thin and clear. The context is brown to rusty brown and becomes black with KOH, with a thickness of 0.5–1.0 cm. The tubules are monolayered, light brown in color, pruinose, and 3–5 mm thick. Their consistency is elastic and flexible in fresh samples, and leathery and rigid in dry ones. They have no smell or taste. The hyphal system is trimitic: the generating hyphae are hyaline, fibulated, with thin walls of 2–4.5 µm in diameter, and are difficult to observe in dried samples. The connective hyphae are quite numerous; the walls range from thickened to solid, brownish yellow, without septa, and they have a sinuous rectilinear shape with a diameter of 3–6 µm. Cystidia and cistidioles are absent. The basidia are clavate-cylindrical and tetrasporic, with basal buckle union, and measure 15–25 (35) × 4–8 µm (Figure 5d). The basiodispores are hyaline, smooth, and cylindrical, sometimes slightly folded up, and measure 7–14 × 3–4.5 (5) µm. They also present notable variations within the same sample. They are essentially saprophytic (less frequently, they are weak parasites) of broad-leaved trees, rarely of conifers on which they cause a very intense and active white rot. The preferred hosts are *Salix* and *Populus*, but they grow on many other substrates. They have been reported on *Quercus cerris*, *Q. suber*, *Fagus sylvatica*, *Populus alba*, *Olea sativa, Acacia cianophylla*, and *Pinus pinea* [36,37].

The genera *Fomitiporia* includes various species, mainly of forestry interest, as agents of wood rot. However, it seems that the *F. mediterranea* species has a preferential relationship with vines. It is more widespread in the Mediterranean area; indeed, it is the only species, to date, of the *Fomitiporia* genus that has been reported not only on vines but also on other plants of agricultural interest (for example, *Olea europaea, Actinidia chinensis, and Citrus* spp.), forestry interest (*Quercus ilex*, *Cornus mas*, *Robinia pseudoacacia*, and *Corylus avellane*), and ornamental use (such as *Lagerstroemia indica*, *Ligustrum vulgare*, *Acer negundo*, and *Laurus nobilis*) [38,39,40]. In the rest of Europe, especially Northern Europe, its presence seems limited exclusively to vines. However, the fungus is capable of fruiting both on vines and on other hosts. In the vineyards of southern Italy, it is difficult to identify the characteristic rust- or cinnamon-colored “crust”; it is easier to find them in old vineyards, particularly on dead plants left standing or “rotting” in piles at the margins of the vineyard. From the pores of these fruitings, which are renewed on the surface every year, the basidiospores spread, resulting in new infections. *Fomitiporia mediterranea* forms resupinate fruit bodies that are woody, 15-mm thick, cinnamon-brown in color, with 6–8 pores per millimeter and an absence of lamellae, generally located on the highest part of the trunk. The basidiospores are of two sizes: 6–7 × 5–6 µm and 5–5.5 × 4–5 µm (but the smaller ones are probably just immature spores). The mycelium is cottony or wooly, with areas of yellowish or brownish hyphae. Some strains (type S, “Straining type”) form sparse aerial hyphae, which confer a dark-red-brown color to the substrate on which they are grown, and they grow more slowly on culture media. The optimal growth temperature is around 30 °C, and reproduction is homothallic [39]. Specific studies conducted in Italy have shown that each infected plant of a vineyard is the result of a new infection [40]. Similar to vines, fungi penetrate olive trees mainly through pruning wounds, colonizing xylem tissues of the trunk and branches, and causing discoloration and wood rot [30,41]. As for enzymatic activity, *F. mediterranea* belongs to a large group of lignicolous fungi that decompose the lignicolous cell walls and cause white rot [31,42,43]. 

*Pleurostoma richardsiae* is a tracheomycotic ascomycete fungus that causes dark streaks in the internal tissues of wood along with necrosis, leaf browning, and phylloptosis. The pathogen has been associated with wood streaks of olive and almond trees in Italy and Spain [10,11] and with vine trunk diseases in Italy, California, and South Africa [7,8,9,12,44]; originally, this fungus was associated with wood streaks of olive trees in Greece. It has also been reported as a pathogen causing olive tree decay in Brazil and Croatia [14]. *P. richardsiae* is also known as a human pathogen and agent of subcutaneous (phaeohyphomycotic) cysts after artificial implants following traumatic events [10].

The *Kirschsteiniothelia* genus was proposed by [45] with *K. etiope* as a species type, characterized mainly by superficial or semi-immersed ascomata, from subglobose to globose, dark brown to black in color, and with cylindrical clubbed, bitunicate, sporated asci. The ascospores are brown to dark brown in color, ellipsoidal, and septate with or without a mucilaginous sheath. The genus has been related to the two anamorphs *Dendryphiopsis* and *Sporidesmium* based on phylogenetic analyses [23]. This genus includes both terrestrial fungi and those capable of colonizing wooden matrices immersed in fresh water [15]. According to [15], this genus consists of 39 species, including *K. nabanheensis*, but most of the species have been identified based on morphological studies. Currently, only 17 species are represented on a molecular basis by DNA in GenBank. *Kirschsteiniothelia* has been reported primarily in the United States (nine species), China (eight species), and Thailand (six species), and little information is available about it being registered in other regions [15]. Therefore, it is unclear whether it is strictly related to specific geographic regions. Therefore, other large-scale investigations are needed in aquatic and terrestrial habitats of different geographic regions and different ecological environments. Further information about hosts and climatic conditions can provide useful information for our more complete knowledge of diversity. 

The artificial inoculation tests carried out using *C. gallica, F. mediterranea*, *Kirschsteiniothelia* sp., and *P. richardsiae* isolates confirmed their pathogenicity in relation to cv. Nocellara del Belice olive trees. It is therefore believed that their presence in decaying trees in the field may be directly related to the described symptoms. Other fungal micro-organisms isolated during the investigations, such as *Alternaria* spp., *A. amstelodami,* and *Trichoderma*, known to be ubiquitous and opportunistic, can be considered only as occasional colonizers. To our knowledge, this is the first report of *C. gallica* and *P. richardsie* on olive trees in Sicily. Furthermore, for the first time, the phytopathogenic capacity of a *Kirschsteiniothelia* isolate was highlighted in *O. europaea*.

The results achieved during this investigation are the first relating to this problem in Sicily in the Trapani Province, and based on these, it is possible to study strategies that can limit or mitigate the syndrome. Our hypothesis is that the irrigation system adopted to obtain valuable table olives, which causes the trunk to become wet in the summer period and, over several decades, may have favored the establishment and development of the aforementioned fungal pathogens. This hypothesis would seem to be confirmed by the progression of decay over time in the trees subjected to the irrigation system described but not reported in olive groves differently managed. The new report of a decay of olive trees in Sicily, thus, seems to be due to both fungal infection and an irrigation system applied by circular collar around the trunk, as described also in *Citrus* trees, where similar irrigation systems are related to damages on branches and trunks and trees show a widespread decay [46]. Probably, the use of different irrigation methods that are not directed at the trunk, yet are still capable of providing adequate quantities of water to produce high quality and quantity levels, would allow the recovery of the less affected trees, albeit not immediately. It is therefore considered appropriate to conduct further and more in-depth investigations aimed at studying the correlation between the irrigation system, presence of fungal agents, and manifestation of the syndrome. A further ongoing investigation is aimed at the use of biostimulants (Agrusaver, Savory Sun, VA LLC) on symptomatic trees, with the aim of both improving the vegetative performance of the host and limiting the symptoms detected in the field.

## Figures and Tables

**Table 1:** Isolation frequency (%) of fungal isolates (number of colonies). Symptomatic trees (small leaves, foliar chlorosis, defoliation, shoot and branch drying, wood rot, cankers, reduced anchoring, flowering, and fruiting) = 1–8; Asymptomatic trees (no symptoms observed) = 9–10. A = apical portion; M = median portion; B = basal portion; *Alt.* spp. = *Alternaria* spp.; *Asp.a* = *Aspergillus amstelodami*; *Cor.g* = *Coriolopsis gallica*; *Fom.m* = *Fomitiporia mediterranea*; *Kir.* sp. = *Kirschsteiniothelia* sp.; *Mic.s* 1 = *mycelia sterilia* (from symptomatic trees); *Mic.s* 2 = *Mycelia sterilia* (from asymptomatic trees); *Ple.r* = *Pleurostoma richardsiae*; *Tri.* sp. = *Trichoderma* sp.; Tot. portion = Total colonies per portion for each tree; Tot. tree = Total colonies per tree.

	Tree 1			Tree 2			Tree 4			Tree 5			Tree 6			Tree 7			Tree 8			Tree 9		Tree 10			% (Number of Colonies)
Taxa	Portion			Portion			Portion			Portion			Portion			Portion			Portion			Portion		Portion			
	A	M	B	A	M	B	A	M	B	A	M	B	A	M	B	A	M	B	A	M	B	M	B	A	M	B	
*Alt.* spp.	1.7 (2)	1.7 (2)								1.7 (2)				0.9 (1)	0.9 (1)	0.9 (1)			0.9 (1)	5.9 (7)							14.5 (17)
*Asp.a*		0.9 (1)								0.9 (1)			1.7 (2)	0.9 (1)	0.9 (1)	0.9 (1)			0.9 (1)	0.9 (1)							8 (9)
*Cor.g*										3.4 (4)		1.7 (2)					3.4 (4)										8.5 (10)
*Fom.m*						1.7 (2)		5.1 (6)										11.1 (13)									17.9 (21)
*Kir.* sp.																					10.3 (12)						10.2 (12)
*Mic.s* 1		1.7 (2)	0.9 (1)	1.7 (2)						1.7 (2)					0.9 (1)	1.7 (2)			1.7 (2)	1.7 (2)	0.9 (1)						12.8 (15)
*Mic.s* 2																							7.7 (9)	0.9 (1)			8.5 (10)
*Ple.r*						6.8 (8)								1.7 (2)	1.7 (2)												10.2 (12)
*Tri.* sp.	1.7 (2)	1.7 (2)								0.9 (1)			1.7 (2)		0.9 (1)	0.9 (1)			0.9 (1)	0.9 (1)							9.4 (11)
**Total port.**	3.4 (4)	6 (7)	0.9 (1)	1.7 (2)	0	8.5 (10)	0	5.1 (6)	0	8.6 (10)	0	1.7 (2)	3.4 (4)	3.5 (4)	5.1 (6)	4.2 (5)	3.4 (4)	11.1 (13)	4.2 (5)	9.4 (11)	11.1 (13)	0	7.7 (9)	0.9 (1)	0	0	**117**
**Total tree**	10.2 (12)			10.2 (12)			5.1 (6)			10.2 (12)			12 (14)			18.9 (22)			24.8 (29)			7.7 (9)		0.9 (1)			

**Table 2:** Results of each fungal strain for the electrophoretic run on agarose gel using different primers.

Fungal Strains	Primers			
	Bt	ITS	EF	CMD
*Coriolopsis gallica*	-	x	-	x
*Fomitiporia mediterranea*	-	x	x	-
*Kirschsteinotelia* sp.	x	x	-	x
*Pleurostoma richardsiae*	x	x	x	x

(Bt = β-tubulin; ITS = Internal Transcribed Spacer; EF = Elongation Factor; CMD = Calmodulin; x = positive; - = negative).

## Data Availability

The original contributions presented in this study are included in the article.
